# New Insights into Fluoroquinolone Resistance in *Mycobacterium tuberculosis*: Functional Genetic Analysis of *gyrA* and *gyrB* Mutations

**DOI:** 10.1371/journal.pone.0039754

**Published:** 2012-06-28

**Authors:** Seidu Malik, Melisa Willby, David Sikes, Oleg V. Tsodikov, James E. Posey

**Affiliations:** 1 Laboratory Branch, Division of Tuberculosis Elimination, National Center for HIV/AIDS, Viral Hepatitis, STD, and TB Prevention, Centers for Disease Control and Prevention, Atlanta, Georgia, United States of America; 2 Department of Medicinal Chemistry, College of Pharmacy, University of Michigan, Ann Arbor, Michigan, United States of America; National Institute of Allergy and Infectious Disease, United States of America

## Abstract

Fluoroquinolone antibiotics are among the most potent second-line drugs used for treatment of multidrug-resistant tuberculosis (MDR TB), and resistance to this class of antibiotics is one criterion for defining extensively drug resistant tuberculosis (XDR TB). Fluoroquinolone resistance in *Mycobacterium tuberculosis* has been associated with modification of the quinolone resistance determining region (QRDR) of *gyrA.* Recent studies suggest that amino acid substitutions in *gyrB* may also play a crucial role in resistance, but functional genetic studies of these mutations in *M. tuberculosis* are lacking. In this study, we examined twenty six mutations in gyrase genes *gyrA* (seven) and *gyrB* (nineteen) to determine the clinical relevance and role of these mutations in fluoroquinolone resistance. Transductants or clinical isolates harboring T80A, T80A+A90G, A90G, G247S and A384V *gyrA* mutations were susceptible to all fluoroquinolones tested. The A74S mutation conferred low-level resistance to moxifloxacin but susceptibility to ciprofloxacin, levofloxacin and ofloxacin, and the A74S+D94G double mutation conferred cross resistance to all the fluoroquinolones tested. Functional genetic analysis and structural modeling of *gyrB* suggest that M330I, V340L, R485C, D500A, D533A, A543T, A543V and T546M mutations are not sufficient to confer resistance as determined by agar proportion. Only three mutations, N538D, E540V and R485C+T539N, conferred resistance to all four fluoroquinolones in at least one genetic background. The D500H and D500N mutations conferred resistance only to levofloxacin and ofloxacin while N538K and E540D consistently conferred resistance to moxifloxacin only. Transductants and clinical isolates harboring T539N, T539P or N538T+T546M mutations exhibited low-level resistance to moxifloxacin only but not consistently. These findings indicate that certain mutations in *gyrB* confer fluoroquinolone resistance, but the level and pattern of resistance varies among the different mutations. The results from this study provide support for the inclusion of the QRDR of *gyrB* in molecular assays used to detect fluoroquinolone resistance in *M. tuberculosis*.

## Introduction

*Mycobacterium tuberculosis* is the etiologic agent of tuberculosis (TB), a potentially fatal illness which results in approximately 2 million deaths worldwide each year [1]. TB treatment requires a lengthy multi-drug regimen, and TB control efforts have been hampered by the emergence of resistance to the first-line drugs. In 2008, approximately 440,000 new cases of TB in the world were resistant to the two most effective first-line drugs, rifampicin and isoniazid (multidrug-resistant TB, MDR TB) [2]. Treatment of patients infected with a drug-resistant strain requires the use of more toxic and less efficient drugs with a longer treatment period as compared to drug-susceptible strains [3]. New, safer drugs are desperately needed to combat the spread of drug resistant *M. tuberculosis*.

The fluoroquinolone (FQ) antibiotics are widely used to treat bacterial infections of the respiratory, gastrointestinal, and urinary tract as well as sexually transmitted diseases and osteomylitis [4]. FQs also have excellent *in vitro* and *in vivo* activity against *M. tuberculosis* and have proven to be among the most effective second-line antimicrobial drugs used for the treatment of individuals infected with MDR TB and patients experiencing severe adverse effects due to first-line drugs [5], [6]. FQs such as moxifloxacin are also being evaluated for use as first-line drugs in treatment protocols designed to shorten treatment duration of drug-susceptible TB [7], [8].

FQs belong to the quinolone class of antibiotics which inhibit bacterial DNA gyrase and topoisomerase IV. DNA gyrase is an ATP-dependent enzyme which cleaves and reseals double-stranded DNA thereby introducing negative supercoils into DNA. This activity is essential for DNA replication, transcription, and recombination [9], [10]. DNA gyrase consists of two GyrA and two GyrB subunits encoded by *gyrA* and *gyrB*, respectively [11], [12], [13]. Topoisomerase IV is also a heterodimer and consists of ParC and ParE subunits encoded by *parC* and *parE*, respectively. Topoisomerase IV relaxes positive supercoils and decatenates DNA following DNA replication allowing the two daughter chromosomes to separate [10], [12]. Many bacterial species possess both DNA gyrase and topoisomerase IV. However, *M. tuberculosis* lacks *parC* and *parE* homologs, and DNA gyrase appears to be the sole target for FQ antibiotics [14].

Despite the potency of FQs in killing *M. tuberculosis*, resistant strains have emerged. FQ resistance in *M. tuberculosis* is mainly due to the acquisition of point mutations within the quinolone resistance-determining region (QRDR) of *gyrA* with codons 90 and 94 being the most mutated sites [15], [16], [17]. Mutations in this region account for 42–100% of FQ resistance in *M. tuberculosis* [16], [18], [19], [20]. Though FQ resistance due to *gyrB* mutations was thought to be rare, clinical isolates resistant to FQs with *gyrB* mutations and wild type (WT) *gyrA* loci were recently reported in several studies [4], [21], [22], [23], [24], [25], [26], [27], [28]. Attempts to understand the contributions of *gyrA* and *gyrB* mutations to FQ resistance have often been carried out by *in vitro* enzymatic assays using purified DNA gyrase [9], [21], [26], [27]. Therefore, the true genetic contributions of some *gyrA* and most *gyrB* mutations to *M. tuberculosis* FQ resistance are not known. To date, functional genetic studies of *gyrA* mutations that are outside the QRDR or *gyrB* mutations in clean *M. tuberculosis* genetic backgrounds have not been undertaken, and certain *gyrA* and *gyrB* mutations reported to confer cross-resistance to different FQ antibiotics based on clinical data have not yet been characterized in well-studied *M. tuberculosis* backgrounds. As a result, the clinical significance of these mutations in *M. tuberculosis* and FQ resistance is unknown. We introduced several mutations identified within *gyrA* and *gyrB* into *M. tuberculosis* laboratory strains and assessed their true significance in FQ resistance. A better understanding of the genetic basis of FQ resistance in *M. tuberculosis* is needed to help in the development of molecular diagnostic tests to rapidly detect drug resistance to ensure TB patients receive a correct treatment regimen.

## Materials and Methods

### Bacterial Strains and Culture Conditions

Plasmids, cosmids and phages used in this study are described in Table S1. *Escherichia coli* DH5α (Zymo Research) and HB101 (Invitrogen) were grown in Luria-Bertani (LB) broth or on LB agar plates at 37°C. *Mycobacterium smegmatis* LR222 [29] and *M. tuberculosis* liquid cultures were grown at 37°C in Middlebrook 7H9 broth supplemented with 10% (vol/vol) albumin-dextrose-catalase (ADC) enrichment (BD Bioscience) and 0.05% (vol/vol) Tween 80 (Sigma). Middlebrook 7H10 agar supplemented with 10% (vol/vol) oleic acid-ADC (OADC) enrichment (BD Bioscience) was used for growing *M. tuberculosis* on solid medium at 37°C. When required, hygromycin B (Invitrogen) was added to growth medium at a final concentration of 150 µg/mL for *E. coli* and 75 µg/mL for *M. tuberculosis*.

### Clinical Isolates

Clinical isolates used in this study (Table 1) were selected from the culture collection of the Laboratory Branch, Division of Tuberculosis Elimination, U.S. Centers for Disease Control and Prevention. These strains had previously been tested for susceptibility to first and select second line drugs using standard procedures (Table S3). FQ susceptibility testing, DNA isolation and sequencing of *gyrA* QRDR was carried out according to methods previously described [15]. In addition, the promoter region and the entire open reading frame (ORF) of *gyrA* and *gyrB* of clinical isolates used in this study were sequenced with primer sets described in Table S2.

**Table 1:** List of *M. tuberculosis* isolates used in this study.

Strain	Background	Mutation		Range of MIC (µg/mL)			
		*gyrA*	*gyrB*	CIP	OFX	LVX	MXF
MLB 135	Clinical isolate	G247S	–	0.5	1	<0.25	<0.25
MLB 5	Clinical isolate	–	V340L	1	1	0.5	<0.25
MLB 105	Clinical isolate	G247S	D500N	2	**4**	**2**	0.5
MLB 159	Clinical isolate	–	D500H	2	**8**	**4**	**1**
MLB 175	Clinical isolate	A384V	M330I	<0.25	0.5	<0.25	<0.25
MLB 20	Clinical isolate	A90V	–	**4**	**4**	**2**	**1**
MLB 263	Clinical isolate	D94G	–	**8**	**8**	**8**	**2**
MLB 264	Clinical isolate	–	N538D+T546M	**4**	**4**	**2**	**1**
MLB 265	Clinical isolate	–	N538T+T546M	2	0.5	0.5	<0.25
MLB 259	Clinical isolate	T80A	–	0.5	0.5	<0.25	<0.25
MLB 261	Clinical isolate	–	R485C+T539N	1–2	**4**–**8**	**2**	**2**–**4**
MLB 262	Clinical isolate	–	N538D	**4**	**4**	**2**	**1**
H37Rv	Laboratory strain	–	–	0.5	0.5	<0.25	<0.25–0.5
Erdman	Laboratory strain	–	–	0.5	0.5	<0.25	<0.25–0.5

−, no mutation, CIP, ciprofloxacin, OFX, ofloxacin, LVX, levofloxacin, MXF, moxifloxacin. Resistance defined as; CIP (>2 µg/mL), OFX (>2 µg/mL), LVX (>1 µg/mL) and MXF (>0.5 µg/mL). Highlighted in bold font are MICs considered resistant to that specific FQ.

### Construction of Recombinant Cosmids Containing Allelic Exchange Substrates

Recombinant cosmids containing allelic exchange substrates were constructed for generating point mutations in *gyrA* and *gyrB* (Table S4). These mutations were introduced into *M. tuberculosis* strains H37Rv and Erdman using the phage delivery system as previously described [30], [31], [32].

A 2.7-kb fragment of *gyrA* was PCR amplified from the genomic DNA of H37Rv using Phusion high-fidelity DNA polymerase (Finnzymes) and the primers gyrAUSF and gyrAUSR (Table S2). The PCR conditions were 98°C for 2 min for denaturing followed by 30 cycles of denaturation at 98°C for 10 sec, annealing at 57°C for 5 sec, extension at 72°C for 45 sec and a final elongation at 72°C for 2 min. The resulting PCR fragment was cloned into pCR-BluntII-TOPO (Invitrogen), yielding plasmid pSM630 (Table S1). To construct plasmid pSM631, a 715 bp PCR fragment of Rv0007, the gene immediately downstream of *gyrA*, was amplified from the genomic DNA of H37Rv using primers gyrADSF and gyrADSR (Table S2) and cloned into pCR-BluntII-TOPO. To construct the recombinant *gyrA* wild-type cosmid pCSM649, the 2.7-kb *Kpn*I/*Xba*I fragment from pSM630 was subcloned into the corresponding restriction sites in pYUB854 [30] upstream of the HYG cassette (Table S1). Furthermore, the 715 bp *Hind*III*/Spe*I fragment from pSM631 containing Rv0007 was cloned into *Hind*III/*Spe*I restriction sites of pCSM649 downstream of HYG cassette to create cosmid pCSM650. The recombinant cosmids pCSM670, pCSM673, pCSM674, pCSM675 and pCSM676 (Table S1) were constructed in the same fashion except that genomic DNA from clinical isolates MLB 259, MLB 20, MLB 263, MLB135 and MLB 175 respectively (Table 1) containing various *gyrA* mutations were used for amplification of the 2.7-kb *gyrA* fragments. Cloned sequences were verified by sequencing. For analysis of *gyrB* sequences, a 2.2-kb fragment was amplified from genomic DNA of H37Rv with primer set gyrBUSF and gyrBUSR (Table S2) using the same PCR conditions as described previously and then cloned into pCR-BluntII-TOPO (Invitrogen) to create plasmid pSM632 (Table S1). In addition, an 830 bp *gyrA* PCR fragment was amplified from H37Rv genomic DNA using primer set gyrBDSF and gyrBDSR (Table S2). The resulting PCR product was cloned into pCR-BluntII-TOPO to yield plasmid pSM633. The recombinant *gyrB* wild-type cosmid was created by subcloning a 2.2-kb *Kpn*I/*Xba*I fragment from pSM632 into the upstream region of HYG cassette in the corresponding restriction sites in pYUB854 resulting in cosmid pCSM651. The 830 bp *Hind*III/*Spe*I fragment cut out from pSM633 (Table S1) was inserted into the corresponding restriction sites downstream of the HYG cassette to create cosmid pCSM652. The recombinant cosmids containing *gyrB* point mutations that were present in clinical isolates of our culture collection were created in the same way as pCSM652 except that, the 2.2-kb fragment for each cosmid was amplified from genomic DNA of clinical isolates as described in Table 1.

### Site-directed Mutagenesis

Cosmids containing points mutations not found in our culture collection of clinical isolates were generated using the QuickChange Lightening site-directed mutagenesis kit (Agilent) with wild-type cosmid pCSM650 (*gyrA*) or pCSM652 (*gyrB*) (Table S1) serving as template and appropriate mutagenic primers described in Table S2. Cosmid pCSM669 containing the double *gyrA* mutation (A74S+D94G) was created using the QuickChange method to insert the A74S mutation into cosmid pCSM674 (D94G). Cosmid pCSM671 (T80A+A90G) was created using a similar approach but with cosmid pCSM670 (T80A) as template. All the constructs were verified by DNA sequencing.

### Construction of Specialized Transducing Mycobacteriophages

The specialized transducing mycobacteriophage used in this study were constructed as previously described [30], [31], [32]. Briefly, the recombinant cosmids (Table S1) were digested with *Pac*I and ligated with phAE159 shuttle phasmid DNA linearized with *Pac*I to generate phasmids. The recombinant phasmids were packaged into phage heads using a λ *in vitro* packaging extract kit (Gigapack III XL, Agilent) and transduced into *E. coli* HB101. Transductants were selected on LB plates containing HYG. Phasmid DNA was isolated from *E. coli* HB101 and electroporated into *M. smegmatis* LR222. The resulting transducing phages were plaque purified and amplified to obtained high titer phage lysate. The presence of allelic exchange substrates was confirmed by PCR and DNA sequencing.

### Transduction of *M. tuberculosis* for the Introduction of Allelic Exchange Substrates

*M. tuberculosis* strains were transduced with high titer phage lysates as previously described [31], [32]. Transductants were selected on Middlebrook 7H10 plates containing HYG and incubated at 37°C for 4 weeks. Point mutations in *gyrA* were verified by PCR and sequencing with primer sets gyrA1F and gyrA1R (Table S2). Two sets of primers were used for *gyrB* point mutations; M330I and V340L were confirmed with primer set gyrB2F and gyrB2R (Table S2), while all other *gyrB* transductants were validated with primer set gyrBSF and gyrBSR (Table S2). The presence of HYG cassette in all the transductants was confirmed by PCR using Hyg-US and Hyg-DS primers (Table S2).

### Structural Modeling of the *M. tuberculosis* Gyrase-DNA-quinolone Complex

Crystal structures of the N-terminal domain of *M. tuberculosis* GyrA (PDB ID: 3IFZ) [33] and the C-terminal domain of *M. tuberculosis* GyrB (PDB ID: 3M4I) [33] were superimposed onto the respective highly homologous regions in the crystal structure of the complex of *Streptococcus pneumoniae* gyrase with a DNA substrate and levofloxacin (PDB ID: 3K9F) [34] by using the SSM superposition tool in Coot [35]. A few minor steric clashes that arose were avoided by minor adjustments of side chain torsion angles. Two residues (R485 and S486) were added to the N-terminus of the C-terminal domain of GyrB in the extended conformation of the backbone to illustrate that R485 is located in the interface with GyrA. This specific backbone conformation is arbitrary, as R485 would be located in the interface with GyrA for any chemically allowed conformation due to the close proximity of the N-terminus of the C-terminal domain of GyrB to this interface.

### Determination of Minimum Inhibitory Concentrations (MICs)

The MICs for ciprofloxacin (CIP) (Sigma), levofloxacin (LVX) (Sigma), moxifloxacin (MXF) (U. S. Pharmacopeia) and ofloxacin (OFX) (Sigma) for *M. tuberculosis* isolates were determined using the agar proportion method as previously described [36]. Clinical strains and transductants were grown on 7H10 agar plates with OADC enrichment containing the following drug concentrations for CIP, LVX, OFX, and MXF: 0.25, 0.5, 1, 2, 4, 8 and 16 µg/mL. Plates were incubated at 37°C and read at 21 and 28 days. The MIC was defined as the minimum concentration of drug which resulted in growth in the drug quadrant that was <1% of that in the no drug quadrant. Resistance to CIP and OFX was defined as MIC >2 µg/mL, LVX, MIC >1 µg/mL, MXF, MIC >0.5 µg/mL (low level resistance) and MIC >2 µg/mL (high level resistance). MIC determinations were done in biological duplicate for each clinical isolate and transductant used in this study to ensure reproducibility.

## Results

### The Significance of *gyrA* Mutations and FQ Resistance

In order to ensure that the MIC observed with clinical isolates harboring mutations in *gyrA* or *gyrB* is directly related to those specific mutations, we introduced single or double mutations into the well-characterized *M. tuberculosis* strains H37Rv and Erdman using the mycobacteriophage allelic exchange system and determined the MIC for CIP, LVX, MXF and OFX. Strains were only considered to be resistant if the MIC for a particular FQ was greater than the recommended critical concentration for the agar proportion method using 7H10 medium [36]. These critical concentration values are 2 µg/ml for CIP and OFX, 1.0 µg/ml for LVX, and 0.5 and 2 µg/ml for MXF. Two concentrations were used for MXF to allow us to discriminate between low and high-level resistant strains.

**Table 2:** MIC of *gyrA* transductants/mutants.

Strain	Background	Mutation	Range of MIC (µg/mL)			
			CIP	OFX	LVX	MXF
A1	H37Rv	WT	<0.25–0.5	0.5	<0.25	<0.25
A2	Erdman	WT	<0.25–0.5	0.5	<0.25	<0.25
A3	Erdman	A74S	1	1–2	1	0.5–**1**
A4	H37Rv	A74S+D94G	**16**	**16**–**32**	**16**	**4**–**16**
A5	H37Rv	T80A	0.5	<0.25	<0.25	<0.25
A6	Erdman	T80A	0.5	0.5	<0.25	<0.25
A7	H37Rv	T80A+A90G	<0.25	<0.25	<0.25	<0.25
A8	Erdman	T80A+A90G	<0.25	<0.25	<0.25	<0.25
A9	Erdman	A90G	<0.25	<0.25	<0.25	<0.25
A10	H37Rv	A90V	2–**4**	2–**4**	0.5–**2**	0.5–**1**
A11	Erdman	A90V	**4**	2–**8**	0.5–**4**	0.5–**1**
A12	CDC1551	D94G	**8**	**8**	**8**	**2**
A13	H37Rv	G247S	<0.25	0.5	<0.25	<0.25
A14	Erdman	G247S	0.5	0.5	<0.25	<0.25
A15	H37Rv	A384V	0.5	1	0.5	<0.25
A16	Erdman	A384V	1	1	0.5	<0.25

WT, wild type. Resistance defined as; CIP (>2 µg/mL), OFX (>2 µg/mL), LVX (>1 µg/mL) and MXF (>0.5 µg/mL). Highlighted in bold font are MICs considered resistant to that specific FQ.

A potential problem with the phage system is the insertion of the hygromycin (HYG) resistance cassette into the chromosome in order to select for the transductants. In this case, the HYG cassette was introduced between the *gyrA* and *gyrB* genes for all transductants. We transduced both H37Rv and Erdman with mycobacteriophage carrying either wild-type *gyrA*/*gyrB* (A1, A2 and B1, B2) or the *gyrA* A90V (A10 and A11) mutation to ensure that the introduction of the HYG cassette did not affect the MIC for the FQs tested in this study. The MIC for CIP, LVX, MXF and OFX of all wild-type transductants was 0.5, <0.25, <0.25 and 0.5 µg/mL (Table 2 and 3), respectively, and these results were similar to the parental strains, H37Rv and Erdman (Table 1). The MIC of transductants carrying the A90V (A10 and A11) allele were also similar to that of a clinical isolate (MLB20) harboring this same mutation (Tables 1 and 2). Based on these results, the phage system is applicable for functional genetic studies of these genes since the introduction of the HYG resistance cassette between the *gyrA* and *gyrB* genes did not influence the MIC of the strains.

**Table 3:** MIC of *gyrB* transductants/mutants.

Strain	Background	Mutation	Range of MIC (µg/mL)			
			CIP	OFX	LVX	MXF
B1	H37Rv	WT	<0.25–0.5	0.5	<0.25	<0.25–0.5
B2	Erdman	WT	<0.25–0.5	0.5	<0.25	<0.25–0.5
B3	Erdman	M330I	<0.25	0.5	<0.25	<0.25
B4	H37Rv	V340L	0.5	0.5	<0.25	<0.25
B5	Erdman	V340L	0.5	1	0.5	<0.25
B6	H37Rv	R485C	1	1	1	<0.25
B7	Erdman	R485C	1	1	1	<0.25–0.5
B10	H37Rv	D500A	0.5	2	1	<0.25–0.5
B11	Erdman	D500A	0.5	2	1	<0.25–0.5
B16	H37Rv	D533A	<0.25	0.5	<0.25	<0.25
B17	Erdman	D533A	0.5	1	<0.25	<0.25
B34	H37Rv	A543T	1	0.5–2	1	<0.25–0.5
B35	Erdman	A543T	1	1	1	<0.25–0.5
B36	H37Rv	A543V	0.5–1	1	0.5–1	0.5
B37	Erdman	A543V	1	2	1	0.5–**1**
B38	H37Rv	T546M	<0.25	0.5	<0.25	<0.25
B39	Erdman	T546M	<0.25	0.5	<0.25	<0.25
B26	H37Rv	T539N	1	2	1	<0.25–0.5
B27	Erdman	T539N	2	2	1	**1**
B28	H37Rv	T539P	1	0.5–1	0.5–1	0.5–**1**
B29	Erdman	T539P	1	0.5–1	0.5–1	0.5–**1**
B24	H37Rv	N538T+T546M	2	0.5	0.5	0.5–**1**
B25	Erdman	N538T+T546M	2	0.5	0.5	<0.25–**1**
B30	H37Rv	E540D	0.5	0.5	0.5	**2**–**4**
B31	Erdman	E540D	0.5–1	0.5–1	0.5	**2**
B22	H37Rv	N538K	2	2	1	**1**–**2**
B23	Erdman	N538K	2	2	1	**1**–**2**
B12	H37Rv	D500H	1–2	**4**–**8**	**2**–**4**	<0.25–0.5
B13	Erdman	D500H	1	**4**–**8**	**2**–**4**	0.5
B14	H37Rv	D500N	1	**4**	**2**	<0.25–0.5
B15	Erdman	D500N	2	**4**	**2**	0.5
B32	H37Rv	E540V	2	**4**	1–**2**	0.5–**1**
B33	Erdman	E540V	2–**4**	**4**	**2**	**1**
B8	H37Rv	R485C+T539N	2	**4**–**8**	**2**–**4**	**2**
B9	Erdman	R485C+T539N	**4**	**8**	**4**	**4**
B18	H37Rv	N538D	**4**	**4**	**2**	**1**
B19	Erdman	N538D	**4**	**4**	**2**	**1**
B20	H37Rv	N538D+T546M	**4**	2	**2**	**1**
B21	Erdman	N538D+T546M	**8**	**4**	**2**	**1**–**2**

WT, wild type. Resistance defined as; CIP (>2 µg/mL), OFX (>2 µg/mL), LVX (>1 µg/mL) and MXF (>0.5 µg/mL). Highlighted in bold font are MICs considered resistant to that specific FQ.

By using this system, we analyzed recently reported mutations (A74S and T80A + A90G) [21], [37] located within the *gyrA* QRDR and two novel mutations identified in this study (G247S and A384V) that are located outside of the QRDR of *gyrA*. The transductants harboring G247S (A13 and A14) and A384V (A15 and A16) exhibited similar MICs as compared to the negative control strains (A1 and A2) and did not confer resistance to any of the FQs tested (Table 2). These results were also similar for clinical isolates containing either G247S or A384V *gyrA* mutations. However, clinical isolate MLB105 which contains a mutation in *gyrA* (G247S) and *gyrB* (D500N) exhibited a higher MIC for all FQs tested (Table 1). The *gyrA* double mutation T80A +A90G (A7 and A8) also had no significant effect on MICs and may actually decrease the MIC for OFX. We also introduced these mutations independently into H37Rv and/or Erdman (A5, A6 and A9) and observed similar results as compared to the double mutation (Table 2). In the case of T80A, the clinical isolate (MLB259) harboring this mutation also has similar MICs.

The A74S mutation (A3) increased the MIC two to four fold for each FQ tested. However, because these values are still below the critical concentration commonly used to test *M. tuberculosis*, this strain is considered susceptible to CIP, OFX, and LVX. This strain exhibited low-level resistance to MXF (MIC of 1 µg/ml) once and susceptible on the repeat test. The A74S mutation has also been reported in combination with a D94G mutation [38]. The single D94G mutation does confer resistance in the clinical isolate (MLB263) and transductant (A12), but the addition of A74S to D94G (A4) further increased the MICs 2–8 fold over that of the single D94G mutation for all FQs tested (Table 2). The combination of A74S and D94G mutations in *gyrA* appears to have a synergistic effect and elevate the MIC for FQs.

### Clinical Relevance of *M. tuberculosis gyrB* Mutations and FQ Resistance

**Figure 1:**
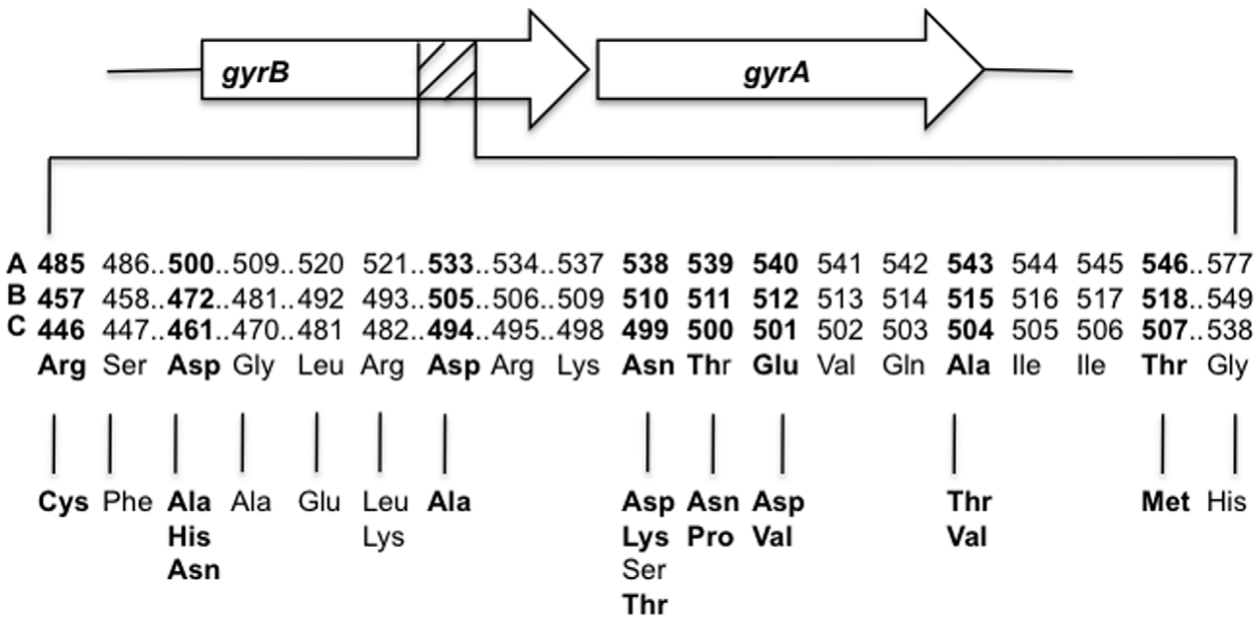
Amino acid substitutions in *M. tuberculosis gyrB*. **A**, numbering system according to http://genolist.pasteur.fr/TubercuList/annotation, **B,** numbering system according to [45] **C**, numbering system according to http://tuberculist.epfl.ch/index.html annotation. Highlighted in bold are the mutations we analyzed in this study.

The presence of *gyrB* mutations, especially at codon D500H and D500N have previously been identified in laboratory strains of *M. tuberculosis* exposed to FQ antibiotics to generate spontaneous FQ resistant mutants [39]. FQ-resistant *M. tuberculosis* strains harboring *gyrB* mutations have been isolated recently from TB patients in several parts of the world, albeit at low frequency [4], [21], [22], [23], [24], [25], [26], [27], [28]. In order to gain better insight into the effects of *gyrB* mutations, we performed functional genetic studies and introduced 19 *gyrB* mutations into pansusceptible *M. tuberculosis* and assessed their role in FQ resistance. At least three different numbering systems based on the choice of the start codon of *M. tuberculosis gyrB* have been used to report the amino acid substitutions in *gyrB* [40]. In this paper, we used the “A” numbering system (Fig. 1) because our experimental design was based on http://genolist.pasteur.fr/TubercuList/annotation. We have also provided the alternative numbering systems in Figure 1 including the one recently proposed by Maruri *et al* [40].

The novel *gyrB* mutations M330I (B3), V340L (B4 and B5), and T546M (B38 and B39) identified in this study did not confer resistance to any of the FQs tested and exhibited MICs similar to the control strains (B1 and B2) with wild-type *gyrB* (Table 3). Transductants harboring the previously published D533A (B16 and B17) *gyrB* mutation [24] were also susceptible to all four FQs. The T539P mutation was identified in an OFX-resistant strain from Vietnam, in combination with an A90V *gyrA* mutation [22]. In a wild type *gyrA* background, the T539P (B28 and B29) mutation alone did not substantially affect the MIC for any of the FQs except LVX (up to 4 fold increase), but the increase of the MIC for LVX was not sufficient to be considered resistant. The A543T and A543V *gyrB* mutations identified in OFX-resistant *M. tuberculosis* strains from Russia [25] were also included in this study. Neither mutation had any effect on the MIC for MXF, but both increased (2–4 fold) the MIC for the other three FQs. Once again, the MICs were still below the critical concentration for this testing method, and the transductants carrying these mutations (B34–37) were considered susceptible to all FQs tested. Based on these data, the M330I, V340L, D533A, T539P, T546M, A543T and A543V mutations are not sufficient to confer resistance to the FQs tested.

The R485C and T539N *gyrB* mutations were reported independently of each other in two studies. The T539N mutation was associated with LVX resistance [28] while the R485C mutation was associated with OFX resistance [23]. The level of cross resistance among other FQs was not investigated in either study. In the present study, these mutations slightly increased the MIC but did not confer resistance to most of the FQs when introduced into H37Rv (B6 and B26) or Erdman (B7 and B27) (Table 3). The T539N mutation did confer low-level resistance to MXF in Erdman. Our results do not agree with the previous reports [28], [41], but in one study, the strains harboring either the T539N or R485C mutation also contained the A90V *gyrA* mutation that is known to confer resistance to FQs [41]. The concentration of LVX used in the other study (1 µg/ml instead of 2 µg/ml) was below the critical concentration for LJ medium [28]. Although, we did not observe these mutations independently of one another in any of our clinical isolates, we identified these mutations together in a single extensively drug resistant (XDR) isolate (MLB 261). This strain was resistant to OFX, LVX, and MXF but susceptible to CIP (Table 1). When we introduced R485C+T539N into H37Rv (B8), this double mutation conferred resistance to OFX, LVX, and MXF, similar to clinical isolate MLB 261 (Tables 1 and 3). However, the same double mutation in the Erdman genetic background (B9) conferred resistance to all of the FQs tested. Based on these results, the individual mutations slightly increase the MIC for each FQ, however, in combination, they act synergistically to increase the MIC above the critical concentration used to test strains by agar proportion on 7H10 media.

Three different amino acid substitutions at codon 500 (D500A, D500H and D500N) in *gyrB* have been reported in *M. tuberculosis* [22], [39], [41]. In one study, the D500H mutation was identified in CIP and OFX-resistant isolates, but the isolates also contained a mutation in *gyrA* known to confer resistance to these FQs [39]. Duong *et al.*, [22] reported clinical isolates harboring the D500N or D500A mutation were resistant to OFX, but the one strain harboring D500A also contained the A90V *gyrA* mutation. We introduced each mutation that occurs at codon 500 into a wild type *gyrA* background to determine if these mutations alone could confer resistance. In this study, transductants harboring D500H (B12 and B13) or D500N (B14 and B15) were resistant to LVX and OFX but susceptible to CIP and MXF (Table 3). These results are also similar for clinical isolates harboring either the D500H (MLB159) or D500N (MLB105) *gyrB* mutation (Table 1). Transductants harboring the D500A (B10 and B11) *gyrB* mutation had an increased MIC for LVX and OFX (at least 4 fold) while the MIC for CIP and MXF was not affected. The transductants are considered susceptible since the MIC is below the critical concentration for each FQ tested. Based on these results, the D500H and D500N mutations confer resistance to OFX and LVX while the D500A mutation does not increase the MIC for any FQ tested above the testing threshold.

Four different amino acid substitutions (N538D, N538K, N538S and N538T) have been reported at codon 538 of *gyrB* in *M. tuberculosis* [4], [9], [21], [22], [25]. The N538D mutation was reported to confer cross-resistance to LVX, MXF and OFX (CIP not tested) in a single clinical isolate, and the gyrase complex containing the mutated *gyrB* was resistant to these FQs in an *in vitro* enzymatic assay [21]. This mutation was associated with resistance to CIP, MXF and OFX but susceptible to LVX in another study [4]. In this study, transductants harboring the N538D (B18 and B19) mutation exhibited cross-resistance to CIP, LVX, MXF and OFX (Table 3). These results were the same for the clinical isolate MLB 262 which also harbors the N538D mutation (Table 1). In addition, the N538K *gyrB* mutation was reported in an OFX-resistant clinical *M. tuberculosis* strain from Russia, but this isolate also harbored the D94G *gyrA* mutation that is known to confer FQ resistance [25]. In this study, the N538K mutation (B22 and B23) exhibited low-level resistance to MXF (MIC 1–2 µg/mL) and increased the MIC level (4 fold) for CIP, OFX, and LVX. However, this increase was not sufficient to be considered resistant to these three FQs.

We also identified two novel double-mutations N538D+T546M and N538T+T546M in clinical isolates MLB 264 and MLB 265, respectively. We have shown in this study that T546M does not confer FQ resistance, and the N538D mutation confers cross-resistance to all the FQs tested. The N538D+T546M mutation (B21) also conferred resistance to all of the FQs tested when introduced into Erdman but did not significantly increase the MIC more than N538D alone. Similar results were obtained for the clinical strain MLB 264 (N538D+T546M) and MLB 262 (N538D) (Table 1 and 3). However, the N538D+T546M double mutation resulted in slightly different results in the H37Rv genetic background (B20) where it was resistant to CIP, LVX and MXF but consistently susceptible to OFX (MIC 2 µg/ml) (Table 3). Transductants carrying N538T+T546M (B24 and B25) mutation were susceptible to all FQs tested which is similar to clinical strain MLB 265 (N538T+T546M). For both the clinical and recombinant strains, the MIC of CIP increased four-fold but remained below the critical concentration. Based on these results, the T546M mutation does not play a synergistic role in FQ resistance, and the N538T mutation does not confer resistance to any of the FQ antibiotics tested. In contrast to this study, the N538T mutation was previously identified in four clinical isolates resistant to OFX, but these clinical isolates also harbored *gyrA* QRDR mutations often associated with FQ resistance [22].

The E540D and E540V mutations were identified in OFX-resistant isolates from Vietnam [22] and were included in this study to assess their clinical relevance. The E540V mutation was recently shown to greatly increase the CIP IC_50_ of the gyrase complex using *in vitro* enzymatic assay [9]. The E540D transductants (B30 and B31) were susceptible to CIP, LVX and OFX but resistant to MXF (MIC 2–4 µg/mL). In this study, the resistance pattern of the E540V mutation was dependent on the genetic background of the transductant strain. In H37Rv, the E540V transductant (B32) was consistently susceptible to CIP but conferred resistance to LVX and OFX and showed a low-level resistance to MXF (Table 3). On the contrary, in the Erdman background, E540V (B33) exhibited cross-resistance to all of the FQs tested during one round of testing but was susceptible to MXF upon repeat testing (Table 3).

## Discussion

FQ resistance in *M. tuberculosis* has been attributed mostly to the modification of the *gyrA* QRDR region with mutations at codon 90 and 94 most commonly associated with drug resistance [15], [16], [17]. Recent studies have identified mutations such as A74S and T80A which are outside of the defined QRDR in clinical isolates alone or in combination with other QRDR mutations, but the effect of mutations outside the traditional QRDR region on FQ resistance is not clear [5]. Although *gyrB* mutations in FQ-resistant *M. tuberculosis* isolates have recently been reported [4], [21], [22], [23], [24], [25], [28], these mutations need to be assessed genetically to enhance our understanding of FQ resistance and possibly improve the molecular testing of FQ-resistant strains.

To date, the best evidence of involvement of *gyrB* mutations in FQ resistance was generated from the use of *in vitro* enzymatic IC_50_ measurements for various FQs toward purified gyrase containing wild type or mutant forms of GyrA or GyrB [9], [21], [26], [27]. Many GyrB mutants exhibit higher IC_50_ values; however it is not known how high the IC_50_ needs to be to confer resistance to FQs. It would be difficult to standardize these assays to allow one to correlate the IC_50_ with the exact MIC for each FQ. Additional evidence for the contribution of mutations within *gyrB* to FQ resistance is based on identification of *gyrB* mutations in phenotypically FQ-resistant isolates [15], [17]. However, it is often difficult to compare FQ-resistant isolates from various studies due to the differences in testing procedures (method and medium for drug susceptibility testing (DST)) and the definitions of resistance. In many cases, the FQ-resistant strains harbored not only *gyrB* mutations but also *gyrA* mutations known to confer resistance and could also contain other unidentified mutations [22], [25].

To circumvent these concerns, we introduced specific mutations into the chromosomal copy of either *gyrA* or *gyrB* of fully susceptible *M. tuberculosis* strains using a specialized phage system and determined the MIC for various FQs. A strain was considered resistant to a FQ if the MIC was greater than the recommended testing critical concentration by the proportion method suggested by CLSI [36]. The allelic exchange system used in this study has been successfully employed by Vilcheze *et al* [32] to transfer *inhA* S94A mutation to *M. tuberculosis* and *M. bovis* BCG to unambiguously demonstrate that S94A indeed confers resistance to isoniazid and ethionamide antibiotics. In addition, Starks *et al* [42] used this same system to prove that *embB* codon 306 confers ethambutol resistance in *M. tuberculosis*. One caveat to the phage system is that the efficiency and preferred site of recombination can vary between constructs. Additionally, the location of the desired mutation within the allelic exchange substrate can also affect the efficiency of its integration into the chromosome. Fortunately, we were able to introduce the desired mutations into multiple genetic backgrounds for most mutations analyzed in this study. However, in a few cases, especially for *gyrA*, this proved more difficult, and we were ultimately unable to introduce a small number of mutations into one or the other background. We did not experience this problem with the *gyrB* mutations probably due to the amount of homologous DNA surrounding the mutations which was much greater as compared to the *gyrA* mutations.

In this study, we identified two mutations, G247S and A384V, located outside of the *gyrA* QRDR in FQ-susceptible and resistant isolates. Neither mutation affected the MIC for any of the FQs tested when transferred into a wild type background. These mutations may be naturally occurring polymorphisms and do not play any role in FQ resistance. In contrast to an earlier report [37], the A74S mutation only slightly increased the MIC for OFX and MXF instead of exhibiting high-level resistance. The discordance between the studies could be due to different genetic backgrounds of the strains, and the clinical strain in the earlier report could possibly harbor additional mutations that also affect the MIC level. The A74S mutation did act synergistically with the D94G mutation and increase the MIC 2–8 fold over either mutation alone. Neither the T80A nor A90G mutation conferred resistance either alone or in combination. In fact, strains harboring the A90G mutation were hypersusceptible to the FQs as demonstrated with *in vitro* enzymatic assays [21]. Thus, with the exception of A74S/D94G combination, mutations outside of the *gyrA* QRDR tested in this study did not lead to FQ resistance.

The QRDR region of *gyrA* is well defined, and the majority of mutations found in this region confer resistance to FQs, albeit not at the same level (unpublished data). The same is not true for *gyrB*, and recently it was proposed to expand the QRDR region of *gyrB* to include amino acids 500–540 (Fig. 1) [27]. More than 15 mutations have been identified in this region alone and several mutations have been identified that are located outside of the QRDR of *gyrB* [40]. The list of *gyrB* mutations continues to grow as more groups analyze *gyrB* of FQ-resistant isolates. Until now, no functional genetic studies in *M. tuberculosis* were completed to definitively determine if a specific mutation was able to confer resistance as determined by standardized DST methods.

We analyzed 19 different mutations located either within or outside of the *gyrB* QRDR to determine their role in FQ resistance. The mutations located outside of the QRDR either had no effect or only slightly increased the MIC levels for the FQs tested. However, this increase was not sufficient to be considered resistant by our testing method. The mutations located within the QRDR of GyrB exhibited an array of MIC levels and conferred resistance to various FQs. D533A was the only mutation located within the QRDR of GyrB that did not exhibit any significant increase in the MIC level for the four FQs. The double mutation, N538T + T546M, was also not sufficient to confer resistance to any of the FQs.

**Figure 2:**
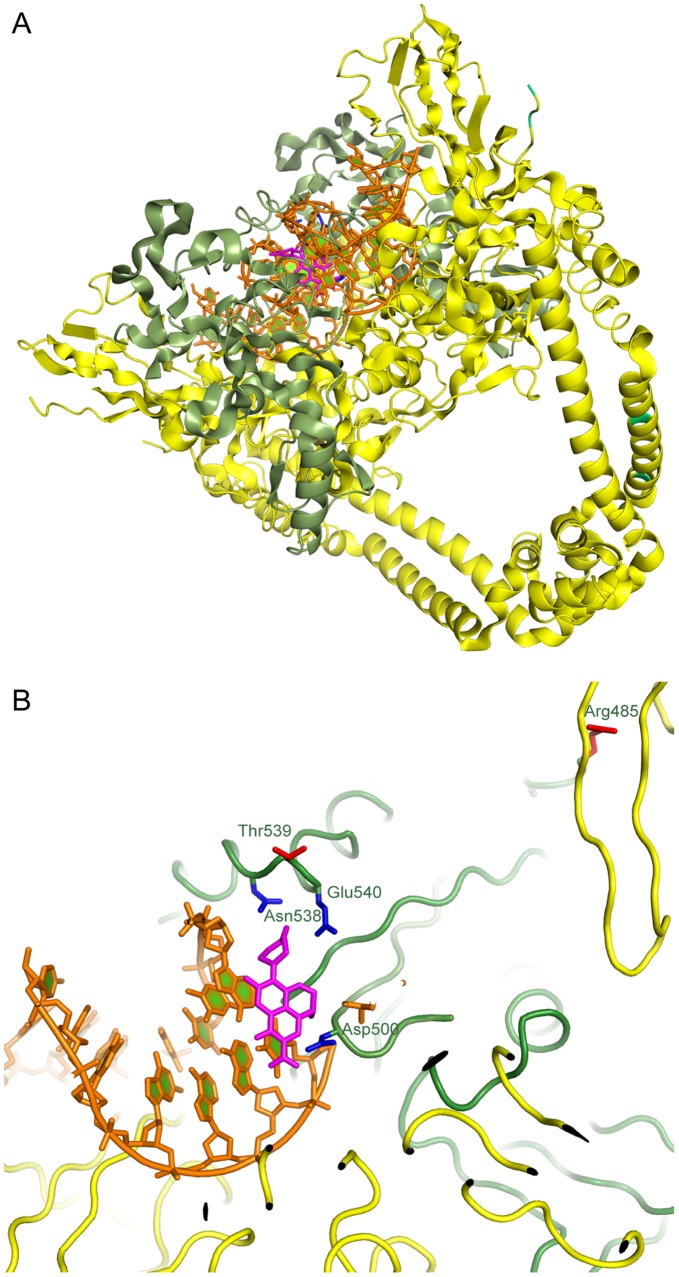
A structural model of *M. tuberculosis* gyrase inhibition. **A.** A model of *M. tuberculosis* gyrase in complex with DNA and levofloxacin. The model was built based on the crystal structure of the complex of *Streptococcus pneumoniae* (PDB ID: 3K9F) as described in Materials and Methods. The GyrA subunit is shown in yellow, GyrB is in green, DNA is in orange, the levofloxacin molecule is shown as pink sticks. **B.** A zoomed-in view of the quinolone binding site. Residues that directly interact with the quinolone and whose mutations cause resistance are shown by blue sticks. The two residues whose double, but not single, mutations cause fluoroquinolone resistance are shown by red sticks.

Several substitutions were analyzed at residues 500, 538, 539, and 540 within the QRDR of GyrB, and different substitutions at a single residue did not confer the same level or pattern of resistance. For instance, the D500A mutation did not confer resistance to any of the FQs tested while D500H and D500N conferred resistance to OFX and LVX. The MIC for OFX and LVX was 2 fold higher for these two mutations as compared to D500A. Based upon the structural model of the *M. tuberculosis* gyrase in complex with a nicked dsDNA substrate and a quinolone in this and other studies [33], [34], D500 lies in the quinolone binding pocket (QBP) and the aliphatic part of this glutamate residue likely interacts with the alkyl or cycloalkyl group at R1 of the FQs (Fig. 2). The carboxyl group of D500 may be involved in hydrogen bonding interactions with the nearby residues and the DNA base that stacks onto the quinolone ring, fitting snugly between the DNA and the drug. The small and nonpolar alanine substitution of D500 would presumably be involved in a similar interaction while being readily accommodating to substitutions as large as a cyclopropyl at R1 of the FQs. In contrast, the larger side chains of histidine and asparagine that bear a positive charge may alter the geometry and electrostatics of the binding pocket and disfavor FQ binding. These mutations conferred resistance to LVX and OFX which have the same R1 group (the active form of OFX is LVX) and did not confer resistance to MXF or CIP which both have a cylcopropyl group at R1.

Residues 538–540 of GyrB form a part of the QBP and interact with the R7 group of FQs. The N538D mutation is one of the more common GyrB mutations found in FQ-resistant isolates [40]. In this study, this mutation conferred resistance to all four FQs tested and increased the MIC levels greater than any other single mutation in *gyrB*. The side chain amine of this asparagine appears to make a hydrogen bond as a donor with the phosphoribose moiety of the nucleotide that stacks with the quinolone ring. In addition, this side chain can also interact sterically with the R7 group in case of a methyl piperazinyl or a comparable sized substitution, fitting snugly between the drug and the DNA. The mutation of this asparagine to an aspartic acid may remove the electron donating character and introduce a rather unfavorable electrostatic repulsion of the DNA backbone thus disfavoring the quinolone binding and resulting in higher levels of resistance. The lysine substitution at this position increased the MIC for all FQs but was only resistant to MXF. The fold increase of the MIC for MXF was greater than the other FQs. The longer side chain of a lysine residue is not easily accommodated in this region, and since the R7 group of MXF is much larger (azabicyclo) compared to the piperazine and methylpiperazine of CIP, OFX and LVX, this may explain why N538K is resistant only to MXF. A threonine at position 538 (N538T) does not alter the electrostatics of the pocket or its geometry as much as the other substitutions and does not lead to resistance for the FQs tested. Based on our modeling and the high structural conservation of this region [34], the side chain of residue T539 points into the solvent; consistently, substitutions at this position (T539N and T539P) did not reproducibly confer resistance. The T539P mutation did confer low-level resistance to MXF for one round of testing. The nonpolar character of the solvent-exposed proline or the higher rigidity of the backbone introduced by this substitution may be structurally perturbing to the backbone, affecting the flanking residues (N538 and E540), both of which interact directly with the drug.

The E540V mutation conferred resistance to all four FQs in this study which was also reported for a clinical isolate with the same mutation in recent studies [9], [27]. This glutamate appears to interact with the drug through hydrophobic interactions between its aliphatic portion and a hydrophobic group at R7 (e.g the methyl of the methyl piperazinyl) whereas the carboxylate likely forms a salt bridge with R521 thereby positioning it for several direct interactions with both DNA and the drug. The valine substitution at this position likely perturbs this arginine coordination. These alterations are sufficient to confer resistance to multiple classes of FQs. Interestingly, the substitution of glutamic acid for aspartic acid (E540D) conferred high-level resistance to MXF and did not significantly alter the MIC for the other three FQs. This substitution may still accommodate the salt bridge with the arginine and would only slightly shorten the side chain, likely affecting interactions with R7. The aspartate residue seems to be able to form hydrogen bonds with CIP, OFX, and LVX which have very similar R7 groups (piperazine and methylpiperazine) but not with the larger R7 group (azabicyclo) of MXF. In line with these structural observations, the effect of the aspartate substitution at this position is relatively subtle.

Mutations located outside of the QRDR of *gyrB* were not capable of conferring resistance alone. However, we identified a unique double mutation, R485C + T539N, with one mutation located within and one outside of the QRDR that conferred resistance to all four FQs, and the MIC levels were usually higher than any single *gyrB* mutation. Based on structural modeling (Fig. 2), the T539N mutation is located within the QBP as described above. The R485C mutation is located at the GyrA-GyrB interface and not near the quinolone binding site of the gyrase complex. The arginine may be important for interactions with GyrA to properly position GyrB relatively to DNA and thus substitutions at this position could affect the quinolone binding pocket allosterically. Together, these two mutations may destabilize the QBP significantly enough to confer resistance. Based on these data, the structural modeling appears to explain the results of the MIC levels and resistance patterns observed for the GyrB mutations investigated in this study.

Based on current literature, up to 58% of FQ-resistant *M. tuberculosis* strains have no identified mutation in the QRDR region of *gyrA* and possibly possess an alternate mechanism of resistance [5], [16], [18], [19], [20]. Factors such as decreased cell wall permeability, active efflux pumps, and drug sequestration or inactivation have been proposed to account for FQ resistance in these isolates [19]. In a previous study, we sequenced the *gyrA* QRDR of 98 FQ-resistant isolates and identified mutations within this region in more than 80% of these isolates. The cause for FQ resistance in the remaining isolates was unknown. Recent publications have suggested a link between mutations within *gyrB* and FQ resistance, and in the present study, we have generated *gyrB* mutations in well-studied genetic backgrounds and demonstrated conclusively that certain mutations within the GyrB QRDR do lead to FQ resistance. Subsequently, we identified FQ-resistance conferring *gyrB* mutations in several of the WT-*gyrA* isolates from our previous study. During the course of the present study we came to appreciate that the level of *gyrA* sequence heterogeneity among specimens isolated from individuals is relatively high with many individuals having both wild-type and mutant *gyrA* sequences. Since, in our hands, Sanger sequencing can only detect sequences that make up greater than 25–50% of the population we chose to enrich for the resistant population in the remaining WT-GyrA, WT-GyrB isolates by growing them on FQ containing media prior to sequencing. Consequently, we identified mutations within *gyrA* in all remaining isolates. Based on these results, we believe that the majority, if not all, FQ resistance in *M. tuberculosis* can be attributed to single nucleotide polymorphisms (SNPs) in *gyrA* and/or *gyrB*. Importantly, several rapid molecular tests have been developed to assess FQ resistance in *M. tuberculosis* utilizing mutations within the GyrA QRDR as markers for FQ resistance [18], [43], [44], and based on the current study, inclusion of the QRDR of *gyrB* in rapid molecular testing which would detect specific substitutions in *gyrB* and *gyrA* would provide a more complete picture of FQ resistance. Unfortunately, FQ resistance imparted by GyrB QRDR mutations seems to be more complex than is the case for GyrA mutations, and the genetic background appears to have some effect on resistance. Molecular assays that analyze the QRDR region of gyrB need to determine the exact mutation since not all mutations confer resistance and the pattern of cross-resistance varies among the mutations.

Most laboratories performing DST for *M. tuberculosis* only test at the critical concentration recommended for their specific testing method. Strains harboring mutations leading to a higher MIC level than wild-type strains but equal to or slightly less than the critical concentration would test susceptible with conventional testing. Data presented in this study demonstrates that this would be the case for many of the *gyrB* mutations. Unfortunately, the importance of these mutations in patient care is unknown. Clinical evidence establishing the efficacy of treatment of individuals infected with strains harboring these types of mutations with various FQ is lacking. However, molecular assays could identify these mutations that result in borderline resistance levels and alert clinicians to possible treatment complications, and in some cases, the genetic information could be useful in tailoring the treatment regimen for the patient.

## Supporting Information

Table S1 **List of plasmids, cosmids and phages used in this study.**
(DOCX)

Table S2 **List of primers used in this study.**
(DOCX)

Table S3 **The drug susceptibility pattern of** ***M. tuberculosis*** **clinical isolates.**
(DOCX)

Table S4 **The nucleotide and corresponding amino acid changes introduced into** ***gyrA*** **and** ***gyrB*** **.**
(DOCX)
