# Supplementary material for: New Insights into Fluoroquinolone Resistance in Mycobacterium tuberculosis: Functional Genetic Analysis of gyrA and gyrB Mutations
Source: PLoS One. 2012 Jun 28;7(6):e39754. doi: 10.1371/journal.pone.0039754 (PMC3386181; doi:10.1371/journal.pone.0039754)
Supplement: Table S1 — List of plasmids, cosmids and phages used in this study. (DOCX) [file pone.0039754.s001.docx]

Table S1. Plasmids, cosmids and shuttle phages used in this study

| Plasmids, cosmids and phages | Characteristics | Source/reference |
| --- | --- | --- |
| Plasmids |  |  |
| pCR-BluntII-TOPO | Vector for cloning blunt-ended PCR products, Kan^r^ | Invitrogen |
| pSM630 | 2.7-kb *gyrA* fragment in pCR-BluntII-TOPO | This study |
| pSM631 | 0.715-kb Rv0007 fragment in pCR-BluntII-TOPO | This study |
| pSM632 | 2.2-kb *gyrB* fragment in pCR-BluntII-TOPO | This study |
| pSM633 | 0.83-kb Rv0006 fragment in pCR-BluntII-TOPO | This study |
| pSM634 | 2.7-kb *gyrA* (*T80A*) fragment in pCR-BluntII-TOPO | This study |
| pSM635 | 2.7-kb *gyrA* (*A90V*) fragment in pCR-BluntII-TOPO | This study |
| pSM636 | 2.7-kb *gyrA* (*D94G*) fragment in pCR-BluntII-TOPO | This study |
| pSM637 | 2.7-kb *gyrA* (*G247S*) fragment in pCR-BluntII-TOPO | This study |
| pSM638 | 2.7-kb *gyrA* (*A384V*) fragment in pCR-BluntII-TOPO | This study |
| pSM639 | 2.2-kb *gyrB* (*M330I*)) fragment in pCR-BluntII-TOPO | This study |
| pSM640 | 2.2-kb *gyrB* (*V340L* fragment in pCR-BluntII-TOPO | This study |
| pSM641 | 2.2-kb *gyrB* (*D500H*) fragment in pCR-BluntII-TOPO | This study |
| pSM642 | 2.2-kb *gyrB* (*D500N*) fragment in pCR-BluntII-TOPO | This study |
| pSM643 | 2.2-kb *gyrB* (*N538D*) fragment in pCR-BluntII-TOPO | This study |
| pSM644 | 2.2-kb *gyrB* (*N538D* + *T546M*) in pCR-BluntII-TOPO | This study |
| pSM645 | 2.2-kb *gyrB* (*N538T* + *T546M*) in pCR-BluntII-TOPO | This study |
| pSM646 | 2.2-kb *gyrB (N538K)* fragment in pCR-BluntII-TOPO | This study |
| pSM647 | 2.2-kb *gyrB* (*R485C*+*T539N*) fragment in pCR-BluntII-TOPO | This study |
| pSM648 | 2.2-kb *gyrB* (*T546M*) fragment in pCR-BluntII-TOPO | This study |
|  |  |  |
| Cosmids |  |  |
| pYUB854 | Used for cloning allelic exchange substrate; contains λ phage *cos* site, Hyg^r^ | [[30](#_ENREF_30)] |
| pCSM649 | *gyrA* fragment upstream of Hyg^r^ cassette in pYUB854 | This study |
| pCSM650 | *gyrA* and Rv0007 fragment flanking Hyg^r^ cassette in pYUB854 | This study |
| pCSM651 | *gyrB* fragment upstream of Hyg^r^ cassette in pYUB854 | This study |
| pCSM652 | *gyrB* and Rv0006 fragment flanking Hyg^r^ cassette in pYUB854 | This study |
| pCSM653 | *gyrA* (*T80A*) fragment upstream of Hyg^r^ cassette in pYUB854 | This study |
| pCSM654 | *gyrA* (*A90V*) fragment upstream of Hyg^r^ cassette in pYUB854 | This study |
| pCSM655 | *gyrA* (*D94G*) fragment upstream of Hyg^r^ cassette in pYUB854 | This study |
| pCSM656 | *gyrA* (*G247S*) fragment upstream of Hyg^r^ cassette in pYUB854 | This study |
| pCSM657 | *gyrA* (*A384V*) fragment upstream of Hyg^r^ cassette in pYUB854 | This study |
| pCSM658 | *gyrB* (*M330I*) fragment upstream of Hyg^r^ cassette in pYUB854 | This study |
| pCSM659 | *gyrB* (*V340L*) fragment upstream of Hyg^r^ cassette in pYUB854 | This study |
| pCSM660 | *gyrB* (*D500H*) fragment upstream of Hyg^r^ cassette in pYUB854 | This study |
| pCSM661 | *gyrB* (*D500N*) fragment upstream of Hyg^r^ cassette in pYUB854 | This study |
| pCSM662 | *gyrB* (*N538D*) fragment upstream of Hyg^r^ cassette in pYUB854 | This study |
| pCSM663 | *gyrB* (*N538D*+*T546M*) fragment upstream of Hyg^r^ cassette in pYUB854 | This study |
| pCSM664 | *gyrB* (*N538T*+*T546M*) fragment upstream of Hyg^r^ cassette in pYUB854 | This study |
| pCSM665 | *gyrB* (*N538K*) fragment upstream of Hyg^r^ cassette in pYUB854 | This study |
| pCSM666 | *gyrB* (*R485C*+*T539N*) fragment upstream of Hyg^r^ cassette in pYUB854 | This study |
| pCSM667 | *gyrB* (*T546M*) fragment upstream of Hyg^r^ cassette in pYUB854 | This study |
| pCSM668 | *gyrA* (*A74S*) and Rv0007 fragment flanking Hyg^r^ cassette in pYUB854 | This study |
| pCSM669 | *gyrA* (*A74S*+*D94G*) and Rv0007 fragment flanking Hyg^r^ cassette in pYUB854 | This study |
| pCSM670 | *gyrA* (*T80A*) and Rv0007 fragment flanking Hyg^r^ cassette in pYUB854 | This study |
| pCSM671 | *gyrA* (*T80A+A90G*) and Rv0007 fragment flanking Hyg^r^ cassette in pYUB854 | This study |
| pCSM672 | *gyrA (A90G)* and Rv0007 fragment flanking Hyg^r^ cassette in pYUB854 | This study |
| pCSM673 | *gyrA (A90V)* and Rv0007 fragment flanking Hyg^r^ cassette in pYUB854 | This study |
| pCSM674 | *gyrA (D94G)* and Rv0007 fragment flanking Hyg^r^ cassette in pYUB854 | This study |
| pCSM675 | *gyrA* (*G247S*) and Rv0007 fragment flanking Hyg^r^ cassette in pYUB854 | This study |
| pCSM676 | *gyrA* (*A384V*) and Rv0007 fragment flanking Hyg^r^ cassette in pYUB854 | This study |
| pCSM677 | *gyrB (M330I)* and Rv0006 fragment flanking Hyg^r^ cassette in pYUB854 | This study |
| pCSM678 | *gyrB* (*V340L*) and Rv0006 fragment flanking Hyg^r^ cassette in pYUB854 | This study |
| pCSM679 | *gyrB* (*R485C*) and Rv0006 fragment flanking Hyg^r^ cassette in pYUB854 | This study |
| pCSM680 | *gyrB* (*R485C+T539N*) and Rv0006 fragment flanking Hyg^r^ cassette in pYUB854 | This study |
| pCSM681 | *gyrB* (*D500A*) and Rv0006 fragment flanking Hyg^r^ cassette in pYUB854 | This study |
| pCSM682 | *gyrB* (*D500H*) and Rv0006 fragment flanking Hyg^r^ cassette in pYUB854 | This study |
| pCSM683 | *gyrB* (*D500N*) and Rv0006 fragment flanking Hyg^r^ cassette in pYUB854 | This study |
| pCSM684 | *gyrB* (*D533A*) and Rv0006 fragment flanking Hyg^r^ cassette in pYUB854 | This study |
| pCSM685 | *gyrB* (*N538D*) and Rv0006 fragment flanking Hyg^r^ cassette in pYUB854 | This study |
| pCSM686 | *gyrB* (*N538D*+*T546M*) and Rv0006 fragment flanking Hyg^r^ cassette in pYUB854 | This study |
| pCSM687 | *gyrB* (*N538T*+*T546M*) and Rv0006 fragment flanking Hyg^r^ cassette in pYUB854 | This study |
| pCSM688 | *gyrB* (*N538K*) and Rv0006 fragment flanking Hyg^r^ cassette in pYUB854 | This study |
| pCSM689 | *gyrB* (*T539N*) and Rv0006 fragment flanking Hyg^r^ cassette in pYUB854 | This study |
| pCSM690 | *gyrB* (*T539P*) and Rv0006 fragment flanking Hyg^r^ cassette in pYUB854 | This study |
| pCSM691 | *gyrB* (*E540D*) and Rv0006 fragment flanking Hyg^r^ cassette in pYUB854 | This study |
| pCSM692 | *gyrB* (*E540V*) and Rv0006 fragment flanking Hyg^r^ cassette in pYUB854 | This study |
| pCSM693 | *gyrB* (*A543T*) and Rv0006 fragment flanking Hyg^r^ cassette in pYUB854 | This study |
| pCSM694 | *gyrB* (*A543V*) and Rv0006 fragment flanking Hyg^r^ cassette in pYUB854 | This study |
| pCSM695 | *gyrB (T546M)* and Rv0006 fragment flanking Hyg^r^ cassette in pYUB854 | This study |
|  |  |  |
| Phages |  |  |
| phAE159 | Temperature sensitive shuttle phasmid derived from the mycobacteriophage TM4 | [[30](#_ENREF_30)] |
| phSM1000 | pCSM650 cloned into unique PacI site of phAE159 | This study |
| phSM1001 | pCSM652 cloned into unique PacI site of phAE159 | This study |
| phSM1002 | pCSM668 cloned into unique PacI site of phAE159 | This study |
| phSM1003 | pCSM669 cloned into unique PacI site of phAE159 | This study |
| phSM1004 | pCSM670 cloned into unique PacI site of phAE159 | This study |
| phSM1005 | pCSM671 cloned into unique PacI site of phAE159 | This study |
| phSM1006 | pCSM672 cloned into unique PacI site of phAE159 | This study |
| phSM1007 | pCSM673 cloned into unique PacI site of phAE159 | This study |
| phSM1008 | pCSM674 cloned into unique PacI site of phAE159 | This study |
| phSM1009 | pCSM675 cloned into unique PacI site of phAE159 | This study |
| phSM1010 | pCSM676 cloned into unique PacI site of phAE159 | This study |
| phSM1011 | pCSM677 cloned into unique PacI site of phAE159 | This study |
| phSM1012 | pCSM678 cloned into unique PacI site of phAE159 | This study |
| phSM1013 | pCSM679 cloned into unique PacI site of phAE159 | This study |
| phSM1014 | pCSM680 cloned into unique PacI site of phAE159 | This study |
| phSM1015 | pCSM681 cloned into unique PacI site of phAE159 | This study |
| phSM1016 | pCSM682 cloned into unique PacI site of phAE159 | This study |
| phSM1017 | pCSM683 cloned into unique PacI site of phAE159 | This study |
| phSM1018 | pCSM684 cloned into unique PacI site of phAE159 | This study |
| phSM1019 | pCSM685 cloned into unique PacI site of phAE159 | This study |
| phSM1020 | pCSM686 cloned into unique PacI site of phAE159 | This study |
| phSM1021 | pCSM687 cloned into unique PacI site of phAE159 | This study |
| phSM1022 | pCSM688 cloned into unique PacI site of phAE159 | This study |
| phSM1023 | pCSM689 cloned into unique PacI site of phAE159 | This study |
| phSM1024 | pCSM690 cloned into unique PacI site of phAE159 | This study |
| phSM1025 | pCSM691 cloned into unique PacI site of phAE159 | This study |
| phSM1026 | pCSM692 cloned into unique PacI site of phAE159 | This study |
| phSM1027 | pCSM693 cloned into unique PacI site of phAE159 | This study |
| phSM1028 | pCSM694 cloned into unique PacI site of phAE159 | This study |
| phSM1029 | pCSM695 cloned into unique PacI site of phAE159 | This study |
