# Supplementary material for: New Insights into Fluoroquinolone Resistance in Mycobacterium tuberculosis: Functional Genetic Analysis of gyrA and gyrB Mutations
Source: PLoS One. 2012 Jun 28;7(6):e39754. doi: 10.1371/journal.pone.0039754 (PMC3386181; doi:10.1371/journal.pone.0039754)
Supplement: Table S2 — List of primers used in this study. (DOCX) [file pone.0039754.s002.docx]

Table S2. Primers used for cloning, site-directed mutagenesis, sequencing and screening of transductants

| Primer sequence |
| --- |
| *gyrA* and *gyrB* cloning primers^a, b^ |
| gyrA USF, 5^'^-GCGGTACCTTGTTCTCCATCCTGATGGG-3^'^ |
| gyrA USR, 5^'^-GCGTCTAGATAGCTGCCCGATTCCTCCTC-3^'^ |
| gyrA DSF, 5^'^-GCGAAGCTTAGGAGGAATCGGGCAGCTAG-3^'^ |
| gyrA DSR, 5^'^-GCGACTAGTCGACCGTGATCATCCAGAC-3^'^ |
| gyrB, USF, 5^'^-GCGGTACCGGGACGCACCAGGAAGAAAG-3^'^ |
| gyrB, USR, 5^'^-GCGTCTAGAGCAGGGTTGCGTTAGACATC-3^'^ |
| gyrB, DSF, 5^'^-GCGAAGCTTGATGTCTAACGCAACCCTG-3^'^ |
| gyrB, DSR, 5^'^-GCGACTAGTAATCCTCTTCTACCTCAACAAC-3^'^ |
|  |
| Site-directed mutagenesis primers^c^ |
| A74S-F, 5^'^-CCACGCCAAGTCG**AGC**CGGTCGGTTGCC-3^'^ |
| A74S-R, 5^'^-GGCAACCGACCG**GCT**CGACTTGGCGTGG-3^'^ |
| A90G-F, 5^'^-CGCACGGCGAC**GGG**TCGATCTACGAC-3^'^ |
| A90G-R, 5^'^-GTCGTAGATCGA**CCC**GTCGCCGTGCG-3^'^ |
| R485C-F, 5'-GCTGGCCGATTGC**TGT**TCCACGGATCC-3' |
| R485C-R, 5'-GGATCCGTGGA**ACA**GCAATCGGCCAGC-3' |
| D500A-F, 5'-TCGTAGAAGGT**GCC**TCGGCCGGCGG-3' |
| D500A-R, 5'-CCGCCGGCCGA**GGC**ACCTTCTACGA-3' |
| D533A-F, 5^'^-GAAAGCGCGCATC**GCC**CGGGTGCTAAAGA-3^'^ |
| D533A-R, 5^'^-TCTTTAGCACCCG**GGC**GATGCGCGCTTTC-3^'^ |
| T539N-F, 5'-GACCGGGTGCTAAAGAAC**AAC**GAAGTTCAGGCG-3' |
| T539N-R, 5'-CGCCTGAACTTC**GTT**GTTCTTTAGCACCCGGTC-3' |
| T539P-F, 5'-CGGGTGCTAAAGAA**CCC**CGAAGTTCAGGCGA-3' |
| T539P-R, 5'-TCGCCTGAACTTC**GGG**GTTCTTTAGCACCCG-3' |
| E540D-F, 5'-GTGCTAAAGAACACC**GAT**GTTCAGGCGATCATCAC-3' |
| E540D-R, 5'-GTGATGATCGCCTGAAC**ATC**GGTGTTCTTTAGCAC-3' |
| E540V-F, 5'-GGTGCTAAAGAACACC**GTA**GTTCAGGCGATCATCA-3' |
| E540V-R, 5'-TGATGATCGCCTGAAC**TAC**GGTGTTCTTTAGCACC-3' |
| A543T-F, 5'-AACACCGAAGTTCAG**ACG**ATCATCACGGCGC-3' |
| A543T-R, 5'-GCGCCGTGATGAT**CGT**CTGAACTTCGGTGTT-3' |
| A543V-F, 5'-CACCGAAGTTCAG**GTG**ATCATCACGGCGC-3' |
| A543V-R, 5'-GCGCCGTGATGAT**CAC**CTGAACTTCGGTG-3' |
|  |
| *gyrA* and *gyrB* ORF sequencing primers |
| GyrA1F, 5^'^-TGGATGTCTAACGCAACCCTG-3^'^ |
| GyrA1R, 5^'^-TTCTCCAGCGCCCAGAACAC-3^'^ |
| GyrA2F, 5^'^-AATATCCCGCCGCACAAC-3^'^ |
| GyrA2R, 5^'^-CAGTGCAATGACCTCGTCC-3^'^ |
| GyrA3F, 5^'^-GGACGAGGTCATTGCACTG-3^'^ |
| GyrA3R, 5^'^-AGCCGCGAATCTGGATGAC-3^'^ |
| GyrA4F, 5^'^-ACCTGTTAGCCTTCCAGC-3^'^ |
| GyrA4R, 5^'^-TTAATTGCCCGTCTGGTC-3^'^ |
| GyrB1F, 5^'^-CCTACGGATAACACGTCGATC-3^'^ |
| GyrB1R, 5^'^-TGAGACCACTCGTACCCGTC-3^'^ |
| GyrB2F, 5^'^-GGTTAACGCGCTATCCAC-3^'^ |
| GyrB2R, 5^'^-TGTTCACCACCGACGTCAG-3^'^ |
| GyrB3F, 5^'^-CCAACACCATCAACACCC-3^'^ |
| GyrB3R, 5^'^-TCGAACTCGTCGTGGATC-3^'^ |
| GyrB4F, 5^'^-GACCGGGTGCTAAAGAAC-3^'^ |
| GyrB4R, 5^'^-TTAGACATCCAGGAACCGAAC-3^'^ |
|  |
| Screening primers |
| gyrA1F, 5^'^-TGGATGTCTAACGCAACCCTG-3^'^ |
| gyrA1R, 5^'^-TTCTCCAGCGCCCAGAACAC-3^'^ |
| gyrB2F, 5'-GGTTAACGCGCTATCCAC-3' |
| gyrB2R, 5'-TGTTCACCACCGACGTCAG-3^'^ |
| gyrBS-F, 5^'^-GTGAACAAGGCTGTGTCC-3^'^ |
| gyrBS-R, 5^'^-CACTTGAGTTTGTACAGCG-3^'^ |
| Hyg-US, 5^'^-CAGGAATTCTGGGAGCCG-3^'^ |
| Hyg-DS, 5^'^-AGTCGTGCAGGAAGGTGAAGG-3^'^ |

^a^Designed based on the sequence of H37Rv [[14](#_ENREF_14)], ^b^restriction enzyme recognition sequences underlined, ^c^the nucleotide changes in the primers to allow A74S, A90G, R485C, D500A, D533A, T539N, T539P, E540D, E540V, A543T and A543V amino acid changes are indicated in bold.
