# Supplementary material for: New Insights into Fluoroquinolone Resistance in Mycobacterium tuberculosis: Functional Genetic Analysis of gyrA and gyrB Mutations
Source: PLoS One. 2012 Jun 28;7(6):e39754. doi: 10.1371/journal.pone.0039754 (PMC3386181; doi:10.1371/journal.pone.0039754)
Supplement: Table S3 — The drug susceptibility pattern of M. tuberculosis clinical isolates. (DOCX) [file pone.0039754.s003.docx]

Table S3. Drug susceptibility pattern of clinical *M. tuberculosis* used in this study

| Clinical strain | RIF | INH | ETH | PZA | CIP | OFX | LVX | MXF | KAN | AMK | CAP |
| --- | --- | --- | --- | --- | --- | --- | --- | --- | --- | --- | --- |
| MLB 5 | R | R | S | S | S | S | S | S | S | S | S |
| MLB 20 | R | R | R | S | R | R | R | R | S | S | S |
| MLB 105 | R | R | R | R | S | R | R | S | R | S | S |
| MLB 135 | R | R | S | S | S | S | S | S | S | S | S |
| MLB 159 | R | R | S | S | S | R | R | R | S | S | S |
| MLB 175 | S | S | S | S | S | S | S | S | S | S | S |
| MLB 259 | R | R | S | S | S | S | S | S | S | S | S |
| MLB 261 | R | R | R | R | S | R | R | R | R | S | S |
| MLB 262 | S | S | S | S | R | R | R | R | S | S | S |
| MLB 263 | S | S | S | S | R | R | R | R | S | S | S |
| MLB 264 | S | S | S | S | R | R | R | R | S | S | S |
| MLB 265 | S | S | S | S | S | S | S | S | S | S | S |

R, resistant S, susceptible, RIF, rifampicin, INH, isoniazid, ETH, ethionamide, PZA, pyrazinamide, CIP, ciprofloxacin, OFX, ofloxacin, LVX, levofloxacin, MXF, moxifloxacin, KAN, kanamycin, AMK, amikacin and CAP, capreomycin. With the exception of PZA, resistance was determined by the agar proportion method on 7H10 medium containing the following drug concentrations: RIF (1µg/mL), INH (0.2µg/mL), ETH (5µg/mL), CIP (2µg/mL), OFX (2µg/mL), LVX (1µg/mL), MXF (0.5µg/mL) KAN (5µg/mL), AMK (4µg/mL) and CAP (10µg/mL). PZA resistance was determined using the BACTEC MGIT 960 at a critical concentration of 100µg/mL PZA.
