# Supplementary material for: New Insights into Fluoroquinolone Resistance in Mycobacterium tuberculosis: Functional Genetic Analysis of gyrA and gyrB Mutations
Source: PLoS One. 2012 Jun 28;7(6):e39754. doi: 10.1371/journal.pone.0039754 (PMC3386181; doi:10.1371/journal.pone.0039754)
Supplement: Table S4 — The nucleotide and corresponding amino acid changes introduced into gyrA and gyrB . (DOCX) [file pone.0039754.s004.docx]

Table S4. Amino acids and nucleotide changes of SNPs in this study

| Locus | Codon | Amino acid change | Nucleotide change |
| --- | --- | --- | --- |
| GyrA | 74 | Ala>Ser | GCC>AGC |
|  | 80 | Thr>Ala | ACC>GCC |
|  | 90 | Ala>Gly | GCG>GGG |
|  | 90 | Ala>Val | GCG>GTG |
|  | 94 | Asp>Gly | GAC>GGC |
|  | 247 | Gly>Ser | GGC>AGC |
|  | 384 | Ala>Val | GCA>GTA |
|  |  |  |  |
| GyrB | 330 | Met>Ile | ATG>ATC |
|  | 340 | Val>Leu | GTG>TTG |
|  | 485 | Arg>Cys | CGT>TGT |
|  | 500 | Asp>Ala | GAC>GCC |
|  | 500 | Asp>His | GAC>CAC |
|  | 500 | Asp>Asn | GAC>AAC |
|  | 533 | Asp>Ala | GAC>GCC |
|  | 538 | Asn>Asp | AAC>GAC |
|  | 538 | Asn>Lys | AAC>AAG |
|  | 538 | Asn>Thr | AAC>ACC |
|  | 539 | Thr>Asn | ACC>AAC |
|  | 539 | Thr>Pro | ACC>CCC |
|  | 540 | Glu>Asp | GAA>GAT |
|  | 540 | Glu>Val | GAA>GTA |
|  | 543 | Ala>Thr | GCG>ACG |
|  | 543 | Ala>Val | GCG>GTG |
|  | 546 | Thr>Met | ACG>ATG |
